# Patient Reactions after the Canterbury Earthquakes 2010-11: A Primary Care Perspective

**DOI:** 10.1371/currents.dis.4ad3beea9e155dd5038a8d2b895f0df4

**Published:** 2014-10-02

**Authors:** Sarb Johal, Zoe Mounsey, Robyn Tuohy, David Johnston

**Affiliations:** Joint Centre for Disaster Research, Massey University / GNS Science, Wellington, New Zealand; Joint Centre for Disaster Research, Massey University / GNS Science, Wellington, New Zealand; Joint Centre for Disaster Research, Massey University / GNS Science, Wellington, New Zealand; Joint Centre for Disaster Research, Massey University / GNS Science, Wellington, New Zealand

## Abstract

Aim – To explore GP perceptions of the impact of the 2010/2011 Canterbury earthquakes on primary care clinic patients. Methods – Qualitative study using semi-structured interviews with eight GPs from the Christchurch area exploring GPs’ perceptions of the impact on patients. Results – Patients experienced significant strain and anxiety following the earthquakes. The impact of this differed due to personal circumstances. Secondary stressors such as insurance and housing issues contributed to experiences of distress. Conclusions – The GPs identified significant impacts on patients as a result of the earthquakes with significant levels of strain and anxiety being due to the on-going recovery process. It appears that a significant proportion of the affected population felt comfortable talking with the GPs about the earthquakes, secondary stressors and their effects upon them.

## Introduction

Experts agree that disasters can have a negative impact on people’s mental well-being^1^. The World Health Organisation estimates that after a disaster, severe mental health disorders increase from 2-3 per cent to 3-4 per cent of the population and mild to moderate mental disorders can double from 10 per cent to 20 percent^2^. However those experiencing mild psychological reactions will be able to cope and recover if they receive support^1^^,^ 3.

Research has also identified that secondary stressors such as lack of financial assistance, insurance issues and continued lack of infrastructure can have an impact on mental well being following a disaster^3^.

Primary care physicians in the community are well placed to deal with the health issues, which commonly present in the post disaster phase^4^. For example, Dorn *et al* (2006)^5^ found that uninjured disaster witnesses increased their number of contacts with primary care doctors by a factor of 1.55 during the first year post-disaster. Within New Zealand, general practitioners (GPs) play a significant role in attending to the health, support and referral of patients who have been affected. The recent patient experience report in New Zealand showed that 84 per cent of adults surveyed expressed confidence and trust in their GP^6^.

On 22 February 2011, Christchurch New Zealand experienced a large earthquake, resulting in the deaths of one hundred and eighty-five people and extensive damage to the built environment. The earthquake was part of a sequence which began in September 2010 and continued with magnitude 5+ earthquakes until December 2011. Aftershocks still occur and are experienced throughout the region.

This research explored GP perceptions of the earthquakes’ impact – both directly and indirectly through secondary stressors - on patient presentations and reactions and provides valuable information to assist in future disaster education, preparation and planning resources for GPs and the local community. Further information on GPs’ experiences in this major New Zealand disaster response contributes to the on-going recovery effort and preparation/planning for future events can be found in a related paper^7^ .

## Methods

A qualitative research methodology was used to explore the GPs experiences and perceptions following the earthquakes. The data were collected between November 2012 and February 2013. Two years had passed since the start of the earthquake sequence, which enabled the research to include longer term impacts and challenges. The research design used semi-structured open-ended interviews to elicit extended answers to questions about the challenges GPs have faced following the earthquakes. The rationale for interviewing GPs was to capture an overview of the health related concerns, which patients presented at primary care clinics. This represents a useful source of additional information when trying to describe the experiences of people going through a disaster and its aftermath, especially those presenting for help in primary care services.

Interviews took place with eight GPs from across the Canterbury area and included practices from different socio-economic areas (poorer to more affluent suburbs). GPs were recruited using a snowball technique in which key informants with knowledge of GP services in the area nominated GPs who were then invited to participate. The interviews were scheduled at a place and time convenient for each GP and audio-taped with permission from each participant. Five female GPs and three male GPs were interviewed covering a range of employment types from locum and salaried GPs to practice director/owner.

Although data proved difficult to obtain, local Primary Health Organisations (PHOs) indicated that 320 GPs worked in the area: Pegasus PHO, 275 GPs (128 female, 144 male, 3 gender unrecorded); Canterbury PHO, 45 GPs. The New Zealand Medical Workforce 2012 data available from the New Zealand Medical Council indicted that 459 GPs were registered in the area, though not all of those may have been practising at the time. This means our study sample somewhere between 1.7% and 2.5% of the regional GP workforce, depending on the denominator.

The transcribed interviews were analysed and coded using a grounded theory approach^8^ . Codes were used to describe common themes that recurred during the interviews. Different members of the research team carried out the data collection and analyses. The data went through several stages of coding and theme generation to understand what the participants saw as significant and important. These themes were checked through discussions within the research team.

The study was peer reviewed and judged to carry low risk of potential harm to participants. This review was recorded on the Massey University Ethics Committee low risk database after having met their set criteria, and participants were informed accordingly.

## Results

The interviews reveal that the earthquakes had a significant impact on GP workload, types of consultations and on patient’s experiences. Four themes were identified in our analysis and are discussed below:

**Impact on workload**

All the GPs talked about the increase in workload they experienced following the earthquake. For some this was due to earthquake-induced population movements. Some areas saw an exodus of people, whereas others saw a significant influx:

“*it was just being busy with normal things because we had too many patients because they were all coming to stay here*”.

However for others the increased workload was due to the number and type of issues presented:

“*a significant amount of that will just be the extra component of emotion, somatic presentation*”.

“saw a lot of patients with acute anxiety initially related to the earthquake”.

GPs reported that patients presented with a range of issues including anxiety, stress, panic reactions, lack of sleep and physical symptoms. Many patients visited their GP “*wanting reassurance*” that what they were feeling was normal. The GPs also commented that two years on from the earthquakes there were still high levels of strain and anxiety but that the cause had changed:

“it’s a different stress...it is actually now coping with the process of thinking in the longer-term”.

“you see people coming in more now and you know they are more stressed about the whole process”.

The GPs recognised that some of the strain and anxiety that patients were experiencing at the time of the interviews was due to the recovery process and focused on housing and/or insurance issues, which:

“was just coming to the fore and the frustration quickly became evident that people weren’t getting EQC [Earthquake Commission] response and insurance response... and that has brought the second major tsunami for people”.

Earthquake damage to residences meant that some of those in rental accommodation had been required to move, possibly to new areas at a time where rental accommodation was in short supply:

“a big problem for some of them is that they have had to move out of their rentals and finding new rentals is...very difficult”.

“a large number of our patients are in rental accommodation or Housing New Zealand [social housing organisation] accommodation”.

Others were living in damaged properties or with relatives waiting for insurance company decisions about repairs:

“a lot of them who have been displaced have moved in with relatives which is causing its own stress...overcrowding, illnesses from a cold house and close contact”.

**Heterogeneous psychological impacts**

Five of the eight GPs talked about how they saw different groups of patients at different times, suggesting that the psychological impact of the earthquake was not evenly distributed both in terms of when patients sought help or who presented for help:

“*first week we saw really anxious stressful people, a week or two later we were seeing other people who we hadn’t seen before with stress and anxiety and not coping...another group about a month or six weeks later which were... generally the rocks and pillars of the community*”.

“actually, people came later on, perhaps several weeks down the track with the more severe symptoms”.

Four of the eight GPs felt that people who had previously experienced mental health issues or who had attended the GP practice following the September 2010 earthquake appeared to cope well:

“it was almost as if their view was ‘one more pile of shit I’ve got to deal with”.

“we didn’t see all the patients who were really, really anxious...after the February earthquake, they coped absolutely fine, they’d learnt how to deal with it I think after the September one”.

Six of the eight GPs commented on the number of children they had seen especially in relation to sleeping disturbance and anxiety:

*“not sleeping, tearful, not wanting to leave the parents, not wanting to sleep in their own room”*.

While initially the sleeping disturbance was probably due to the significant number and strength of the aftershocks, the GP comments from their own personal experiences suggested that this may be an ongoing issue:

“it was the children not sleeping...for two years she had to have someone lie down next to her to sleep”.

One GP commented specifically about stressed nine to twelve year old boys:

“*they were probably the only kids I had to refer to anybody*”.

Three of the GPs talked about the impact on their elderly patients, with particular concern for those patients who lived alone:

“the elderly are particularly challenged, they’d been prepared for retirement, paid off mortgages, had their families set up to support them and it’s been pulled from under them”.

“*this just really upset the applecart and it started presenting as memory loss, you know so much high stress, so much going on, just can’t remember what they did and they were just in a state of almost panic all the time*”.

Other patient groups such as migrant and refugee populations with complex health and psychological needs were significantly affected following the earthquake as the group of people requiring housing support increased:

“there are not only less houses but that there is more people in need”.

**Support**

The GPs acknowledged the support that was put in place to help patients in the form of access to counselling services and government funded extended consultations with the GP (ordinarily in the NZ health care system the majority of patients make a co-payment for GP appointments):

“great resources for those people who have needed extra counselling for the anxiety”.

“that was probably one of the services that was the most valuable to the community, just to be able to access that without the cost involved”.

“it was a real honour to be able to provide that service”.

These additional services helped the GPs to deal with the increased workload, and the widespread communication programme concerning the availability of mental health assistance combined with ease of co-located access to counselling meant that patients did not feel stigmatised accessing the service:

“One of the patients said “no stigma, you know, I am not going to a mental health clinic” ”.

**Community spirit**

The GPs talked about the community spirit that was evident following the earthquakes both in terms of people helping to get GP practices up and running and in terms of the wider community:

“a lot of people coming and going with boots, and shovels and wheelbarrows started arriving...helping to clear up”.

“you saw a lot of that, you know, camaraderie and really people in Christchurch became a lot more community spirited”.

“all these people helping and baking and just you know, the armies of people going and digging silt for strangers”.

There was a sense from some of the interviews that has time has gone by this community support has dissipated and people are now at different stages of the recovery process:

“now it’s like some people’s lives are back to normal and others are still hanging on the edge and yet perhaps they are not getting the support as much as what they were in the early days”.

“it’s like some people’s lives are back to normal and others are still hanging on the edge”.

## Discussion

The GP interviews indicate that patients experienced stress and anxiety as a result of the earthquakes, though the impact of this differed depending on individual patients’ circumstances. Many patients just wanted to talk through their experience and get reassurance that their feelings were normal. Interestingly some GPs identified ‘waves’ of different patient groups consulting at different times with higher levels of psychological distress coming some weeks after the earthquake. This could reflect people moving through the early phases of the disaster which have been termed ‘heroic’ and ‘honeymoon’ phases^9^^,^ 10. Raphael (1986)^10^ described the honeymoon period as a time during which survivors benefit from a wave of compassion, goodwill and care. This also fits with the rise in community spirit identified by the GPs. However, this level of response cannot be sustained and there often follows an “*emotional trough during which disillusionment sets in as the survivors wrestle with what they regard as bureaucratic and legal barriers*” (Alexander, 2005, p14). Another aspect of this disaster was the continuing aftershocks which meant that people experienced on-going acute stress after the initial disaster^9^ .

The GPs also identified particular demographic groups that had been affected by the earthquake such as children and the elderly. Previous research^11^^,^ 12 has identified that children are particularly vulnerable to the effects of disasters. Children may experience multiple stressors during the disaster as well as months and years afterwards, including loss of home and personal property, relocation, change of school, loss of friends and pets and disruption of family and community resources^12^ . These can result in numerous psychological and physiological stress reactions^13^. The most common issues for children reported by the GPs in the interviews were sleep problems and not wanting to be left alone.

The GPs described some of the impacts on their elderly patients. Research has reported that disasters can result in increased vulnerability of older people particularly greater mortality, decline of physical health, functional and cognitive decline, increased emotional distress and loss of social supports^14^^,^ 15 ^,^ 16.

While our findings relating to children and elderly patients are broadly in line with previous research, GP commentary relating to patients with previous experience of mental ill health differed from previous research. Freedy & Simpson (2007)^4^ suggested that those with on-going mental health concerns are at high risk following a disaster. However, several GPs in this study reported that patients with prior experience of mental health issues coped better than expected. The closeness of the two high magnitude earthquakes may have resulted in the successful development and use of coping strategies for some patients. Previous experience of adverse circumstances and struggles to maintain mental wellbeing could have acted as a protecting influence and resource to draw upon. Additionally, increased levels of community support may also have contributed to better coping. Indeed, an integrative approach to PTSD put forward by Foy at al (1993)^23^ proposes that other factors may mediate between trauma exposure and the conditioning of acute or chronic symptoms. These psychological , social and biological factors may be considered as risk factors when their presence increases the risk of symptomology, or resilience factors when their presence decreases the likelihood of symptoms emerging. In this case, what appears to be reported is that previous experience of mental health difficulties did not act as a risk factor, but instead was a relatively benign factor, if not an outright resilience source for those people identified by these GPs.

The concept of emotional work or emotional labour may also be useful here (Hochschild, 1979, 1983)^25^^,^ 26 . This work relates to the ways in which “the individual ... works on inducing or inhibiting feelings so as to render them ‘appropriate’ to a situation” (p. 551). In this case, those patients in this study with previous mental health difficulties already have a model of the emotional work or labour available to them, or ‘conventions of feeling' (Whittle et al, 2012)^27^ , and as such are better prepared to undertake the processing of the dramatic situation that has unfold in their lives.

It is encouraging that people appear able to present to their primary health care provider for help after disaster, albeit in waves. Souza, Yasuda and Cristophani (2009)^17^ talk about the integration of mental health care into primary care systems in humanitarian emergencies. The reduction of the potential for stigma of being referred to a mental health service and being assisted within the primary care setting also appears to be helpful in this disaster response and recovery context. This can be particularly helpful for patients presenting with somatic complaints that may have no apparent physical etiology.

Recent research about post-disaster recovery suggests that the post-disaster stressors, such as delayed decisions about property and insurance, are some of the most significant risk factors for mental ill health^18^. These ‘secondary stressors’ are circumstances, events or policies that are indirectly related or ‘non-inherent and consequential’ to the earthquakes^19^. Examples include housing difficulties, problems with insurance and loss of social networks; these can manifest their effects shortly after a disaster and persist for extended periods of time^3^. These interviews indicate that patients were continuing to experience high levels of stress and anxiety as a result of ongoing housing and/or insurance issues. The GPs voiced concern about the impact of this stress and anxiety moving into the future.

The Canterbury Earthquake Recovery Authority Wellbeing Survey^20^ acknowledges some positive outcomes as a result of the earthquakes, in particular ‘heightened sense of community’. The GP interviews also illustrate the high levels of community spirit and support that followed the earthquakes. However the findings of research conducted by the ‘All Right?’ campaign^21^ and a Christchurch Earthquake Recovery Authority Wellbeing Survey^22^ indicate that Christchurch residents continue to experience negative effects as a result of the earthquakes. In September 2012, over half of Christchurch residents believed that their quality of life had deteriorated since the earthquakes^20^ . In the most recent Wellbeing Survey twenty three percent felt that their quality of life had deteriorated in the past 12 months^22^. Although the proportion of people who reported that decreased quality of life had reduced, seventy eight per cent of residents still reported experiencing stress in the past twelve months that had had a negative effect on their lives^22^. The three most prevalent issues that were impacting on everyday life were dealing with Earthquake Commission (EQC) and/or insurance issues, making decisions about house damage, repairs, and relocation and being in a damaged environment and/or surrounded by construction work^22^. A key message from qualitative research undertaken as part of the ‘*All Right?*’ campaign suggests that the earthquakes have been seen as a ‘double blow’ to some – the earthquakes themselves and the perceived poor management of the recovery^21^ . This was reflected in the interviews in this research as GPs acknowledged that people were at different phases of the recovery process, with some seemingly getting on with their lives and others still “*hanging on the edge*”.

## Implications

Our research has shown that GPs continue to play a vital support role within the community during the post disaster recovery of Christchurch and are an important source of information. This role has also been confirmed in findings from the All Right? Becoming All Right? (2013) survey^21^ conducted in the Christchurch region, which found respondents listed their GP or doctor to be in the top five people/groups identified in their support network. Therefore, knowledge of the post disaster primary care challenges that GPs faced in caring for their communities, is a valuable source of learning and future planning for primary health care practice.

**Table 1: Lessons Offered:** 

GPs identified significant strain and anxiety in patients presenting at primary care clinics, which accounted for a large proportion of their increased workload after the Canterbury earthquakes.
This increased distress seemed to stem from multiple causes, including fear of recurring earthquakes and ongoing issues due to the impact of secondary stressors.
GPs reported children, older adults and migrants / refugees as groups that seemed to be disproportionately affected when attending at primary care.
Half of the GPs interviewed commented that those with previous mental health issues or who had presented following the September 2010 earthquake appeared to cope better with subsequent earthquakes.
Although community spirit seemed to increase in the immediate aftermath of the earthquakes, this appeared to dissipate as different parts of the community moved through the recovery at different speeds.
GPs reported that the extra resources made available (counseling services and extended consultation time) helped them to cope with their increased workload, and were appreciated and well-used by patients.
